# Imaging Biomarkers as Predictors for Breast Cancer Death

**DOI:** 10.1155/2019/2087983

**Published:** 2019-04-10

**Authors:** Wendy Yi-Ying Wu, Laszlo Tabar, Tibor Tot, Ching-Yuan Fann, Amy Ming-Fang Yen, Sam Li-Sheng Chen, Sherry Yueh-Hsia Chiu, May Mei-Sheng Ku, Chen-Yang Hsu, Kerri R. Beckmann, Robert A. Smith, Stephen W. Duffy, Hsiu-Hsi Chen

**Affiliations:** ^1^Department of Radiation Sciences, Oncology, Umeå University, Sweden; ^2^Department of Mammography, County Hospital Falun, Falun, Sweden; ^3^Department of Pathology, Laboratory Medicine Dalarna, County Hospital Falun, Falun, Sweden; ^4^Kainan University, Taiwan; ^5^Taipei Medical University, Taiwan; ^6^Chang Gung University, Taiwan; ^7^National Taiwan University, Taipei, Taiwan; ^8^University of South Australia, Adelaide, Australia; ^9^American Cancer Society, Atlanta, GA, USA; ^10^Queen Mary University of London, UK

## Abstract

**Background:**

To differentiate the risk of breast cancer death in a longitudinal cohort using imaging biomarkers of tumor extent and biology, specifically, the mammographic appearance, basal phenotype, histologic tumor distribution, and conventional tumor attributes.

**Methods:**

Using a prospective cohort study design, 498 invasive breast cancer patients diagnosed between 1996 and 1998 were used as the test cohort to assess the independent effects of the imaging biomarkers and other predictors on the risk of breast cancer death. External validation was performed with a cohort of 848 patients diagnosed between 2006 and 2010.

**Results:**

Mammographic tumor appearance was an independent predictor of risk of breast cancer death (P=0.0003) when conventional tumor attributes and treatment modalities were controlled. The casting type calcifications and architectural distortion were associated with 3.13-fold and 3.19-fold risks of breast cancer death, respectively. The basal phenotype independently conferred a 2.68-fold risk compared with nonbasal phenotype. The observed deaths did not differ significantly from expected deaths in the validation cohort. The application of imaging biomarkers together with other predictors classified twelve categories of risk for breast cancer death.

**Conclusion:**

Combining imaging biomarkers such as the mammographic appearance of the tumor with the histopathologic distribution and basal phenotype, accurately predicted long-term risk of breast cancer death. The information may be relevant for determining the need for molecular testing, planning treatment, and determining the most appropriate clinical surveillance schedule for breast cancer patients.

## 1. Introduction

The first generation of breast cancer prognostic indicators, specifically tumor size, histologic malignancy grade, and spread to lymph nodes have long influenced treatment regimens [1–4]. However, these conventional tumor attributes may not be sufficient for predicting the prognosis of breast cancer after the era of early detection via the wide spread use of mammography and molecular tumor subtypes. The mammographic appearance, which reflects pathological changes of breast anatomy, has been reported to be a reliable, independent imaging biomarker for breast cancer death [5–9]. Six subtypes have been classified based on the features of tumor mass and calcifications [10]. In addition to making use of radiological morphology, over the past decade molecular markers and gene expression profiling have been considered a new set of prognostic indicators. The first molecular breast cancer biomarkers with predictive value were described by Perou [11] and since then additional markers have been identified [12–16]. The most interesting marker may be the basal phenotype, which reportedly is related to the* BRCA*1 mutations and has been shown to be associated with unfavorable prognosis [16, 17]. The early detectability of breast cancer is further enhanced by assessing the extent of breast tumors determined by large-section histopathology [5, 6, 18–22]. In particular, the distribution of the breast cancer foci, determined by large-section histology, has been advocated to differentiate between breast cancer with a good prognosis (unifocal tumors) and breast cancer with poorer prognosis (multifocal and diffuse breast cancers) [23].

Information on mammographic appearance, tumor phenotype, and histological tumor distribution together with conventional tumor attributes is readily available in countries with organized mammography screening programs, and these features are highly relevant where a significant and growing fraction of patients are diagnosed with small, favorable prognosis tumors. A better understanding of long-term prognostic indicators associated with small tumors is desirable to avoid overtreatment, but also to ensure appropriate treatment in the small percentage of patients with small tumors whose poor prognosis presently is not well predicted by conventional tumor attributes [24]. The tumor's appearance on the mammogram, a low resolution black, and white image of the underlying histopathology can contribute additional useful information for treatment planning when combined with the molecular characteristics of tumors.

The future of individualized medicine depends on continued progress in risk profiling based on new tumor markers and other useful disease features and on identifying the clinical circumstances when various combinations of these data are useful for treatment planning. How to identify the complementary role and relevance of each factor with the others in predicting the risk of breast cancer death is of paramount important. For instance, the clinical outcome of the two most fatal breast cancer subtypes, as determined by radiological morphology (architectural distortion without calcification, and casting type calcifications) is not predictable using existing biomarkers [5, 6]. The highly fatal diffuse breast cancers appearing on the mammogram as architectural distortion are generally moderately differentiated cancers, most of which are ER/PR positive, HER2 negative, with low proliferation index (1-10% Ki67). In cases with casting type calcifications on the mammogram and an associated small invasive cancer, the biomarkers are unfortunately determined only on the small invasive cancer tissue rather than on the neoductgenesis represented by the casting calcifications [5].

The aim of the present study is to explore whether imaging biomarkers such as the mammographic appearance of the tumor, the tumor phenotype with particular emphasis on basal phenotype, and the histologic tumor distribution (unifocal, multifocal, and diffuse) can be combined to differentiate risk groups for breast cancer death in a longitudinal cohort of women diagnosed with breast cancer that was derived from a population-based organized mammography screening program.

## 2. Materials and Methods 

### 2.1. Study Subjects and Data Collection

We identified 1,346 consecutive patients diagnosed with primary invasive breast cancer at Falun Central Hospital, Sweden, of which 498 were diagnosed between 1996 and 1998 and 848 were diagnosed between 2006 and 2010. A prospective cohort study design was adopted by treating the former series as the test cohort and the latter series as the validation cohort. The predictors considered were based on the mammographic appearance of the tumor, basal phenotype, and the histologic tumor distribution together with the conventional tumor attributes (tumor size, axillary node status, and histologic malignancy grade). Note that as type of surgery and adjuvant therapy are driven by these correlated but complementary features, they are regarded as controlled variables when the respective independent effects of each factor on the survival of breast was assessed. Date and cause of death were obtained from the Swedish National Death Registry.

Breast cancer care at Falun Central Hospital has been coordinated by a multidisciplinary tumor board including radiologists, pathologists, breast cancer surgeons, and oncologists to review the screening mammogram, confirmatory diagnosis by ultrasound, MRI, and other alternative imaging studies, pathological reports based on large-section method, and the choice of treatment modalities. Information on mammographic appearance of the tumor, including tumor distribution and extent, was available to a preoperative tumor board for each case. After excision, with the aid of whole specimen radiograph, the most appropriate sections were obtained to represent the proper extent of the breast lesion. Each case was discussed by the postoperative tumor board, which verified the concordance of radiologic and histologic findings. The three families of predictors were determined as follows.

### 2.2. Mammographic Appearance of the Tumor

The classification system developed by Tabár was used to assess mammographic tumor appearance [6, 25]. Six mammographic categories were used, each having histologically proven invasive carcinoma: (1) stellate mass with no associated calcifications; (2) circular mass with no associated calcifications; (3) calcifications of crushed stone-like appearance, with or without associated tumor mass; (4) powdery calcifications, with or without associated tumor mass; (5) casting type calcifications, with or without associated tumor mass; and (6) architectural distortion.

### 2.3. Molecular Classification

Immunohistochemical assessment was made using the methods for molecular phenotypes given by Pekar et al. [26]. Tumors expressing at least one basal (myoepithelial) marker (CK5/6, CK14, or EGFR) were categorized as basal phenotype tumors [26]. Other markers such as HER-2, ER and PR status were used to compare various classification systems (see supplementary Tables S1-S6).

### 2.4. Tumor Attributes, Histologic Tumor Distribution, and Treatment Modalities

The details of the determination of these pathological features have been fully described elsewhere [21, 23]. Briefly, a team of four pathologists reviewed all large-format histology slides without having information regarding the mammographic tumor features. The 1996-1998 material was reviewed at a meeting of the four pathologists on our breast team. They analyzed the subgross features on an overhead monitor and the microscopic features in a multiheaded microscope. The pathohistological parameters in the original pathology report were either confirmed or modified following the consensus in the group. The 2006-2010 material was reviewed by three pathologists. Histologic tumor distribution was classified as unifocal, multifocal, or diffuse. Representative tumor areas were identified for taking punch biopsies to determine the phenotype of the tumor with immunohistochemistry. In unifocal carcinomas with heterogeneous histologic appearance, as well as multifocal and diffuse cancers, multiple punch biopsies were obtained. In addition to histologic tumor distribution, conventional tumor attributes (tumor size, node status, and histologic malignancy grade) were also determined from the large-section histology [23]. Tumor size was categorized as a binary variable: 1-14 mm or ≥ 15 mm. Axillary lymph node involvement was classified as positive or negative. Histologic malignancy grade was also determined and classified as well-differentiated, moderately differentiated, or poorly differentiated following the Bloom-Richardson system as modified by Elston and Ellis [27]. Note that these three pathologists in the late period were the same members in the original team between 1996 and 1998. The infrastructure of this department of pathology is very stable with time and the consistency between two cohorts in terms of operational viewpoint on pathology is high. For example, the large-section method for detecting histological tumor distribution (such as unifocal, multifocal, and diffuse) and microarray analysis has been adopted by this team since 1996.

### 2.5. Statistical Analysis

We assessed the associations between pairs of predictors in the two cohorts combined using the chi-squared test. The relative contribution of each predictor to the risk of breast cancer death was determined based on Cox proportional hazards regression analysis of the first series of 498 patients (diagnosed between 1996 and 1998) who were followed until death or December 31, 2010. Survival time was computed from the date of diagnosis until death or the end of the study. Breast cancer death was treated as an uncensored event, whereas other causes of death were regarded as censored events. The survival curves by mammographic appearance of the tumor, basal phenotype, and histologic tumor distribution were plotted using the life-table method.

In addition to the descriptive analysis indicated above, the present study has two main analyses for deriving informative analytic results. The first analysis is to assess the respective independent effects of each relevant variable on the survival of breast cancer. We estimated the hazard ratios (HRs) for mammographic appearance, basal phenotype, histologic tumor distribution, conventional tumor attributes, type of surgery, and adjuvant therapy. In the multivariable regression model, we treated tumor size, node status, histologic malignancy grade, type of surgery, and adjuvant therapy as established predictors that are retained in the basic model although these variables are correlated with or driven by the imaging biomarkers of interest. After first incorporating the mammographic appearance of the tumor, we observed several issues of collinearity when we added basal phenotype and histologic tumor distribution to assess whether they were prognostic predictors independent of the tumor's mammographic appearance. For example, the stellate, powdery calcifications, and crushed stone-like calcification appearances were rarely seen in combination with the basal phenotype (see below). We therefore combined these three categories in order to produce concise information, since previous results suggested that they all have similar prognosis [10]. Because of the similarity of the prognosis, the clinical treatment and therapies are driven by imaging biomarkers in the same way that certain therapies are only prescribed for specific molecular tumor subtypes (e.g., adjuvant hormone therapy would only be prescribed for hormone sensitive tumors).

The second analysis is focused on producing a new classification of risk groups based on empirical survival estimates before the administration of treatment and adjuvant therapies and the selected variables with reference to the results of the Cox proportional hazards regression model and the cumulative survival curves. We classified the three-dimensional variables (mammographic appearance of the tumor, basal phenotype, and histologic tumor distribution) into 12 risk groups. The 10 yr risk of breast cancer death and the 95% confidence interval directly using the test cohort data are presented. Note that the order of predictors used for the new classification is not based on statistical criteria but pursuant to strong evidence from the chronological order of acquired clinical information, beginning with the finding of mammographic features, the immunochemical test with emphasis on basal phenotype, large-section pathological findings, and tumor size, all of which led to the decision of surgical type and adjuvant therapy.

To validate the proposed model, we estimated regression coefficients (clinical weights) for each group, with adjustment for conventional tumor attributes, type of surgery, and adjuvant therapy, using the Weibull accelerated failure time model [28]. We predicted the risk of breast cancer death by applying the estimated regression coefficients to the validation cohort which was followed until Dec. 31, 2015. As these patients were diagnosed with breast cancer between 2006 and 2010, the regression coefficients estimated from the Weibull accelerated failure time model were adjusted for the baseline hazard rate corresponding to the underlying rate of breast cancer death for the period between 2006 and 2015.

As far as this validation procedure is concerned, we follow the traditional paradigm that divides the entire dataset into the trained dataset and the validation dataset. The only difference is that the decision influencing the selection of each dataset is based on their chronological order with external validation, rather than the division of the entire cohort into the test cohort and the validation cohort with cross-validation, because there was interest in examining whether imaging biomarkers identified in the early period of digital mammography (1996-1998) can be applied to the latest period (2006-2010). The test dataset was used to estimate the weights, namely, the regression coefficients, contributed from each predictor of these emerging imaging biomarkers, tumor phenotypes, and conventional tumor attributes that have been well recognized from numerous previous clinical studies without doing model selection. The effect sizes of these regression coefficients were further applied to the validation dataset to estimate the expected values in comparison with the observed one to check the adequacy of the proposed predicted risk model using a chi-squared test. We also compared the performance of the model with the incorporation of imaging biomarkers with one that only used conventional tumor attributes by using the area under the curve (AUC) of the receiving operating characteristics (ROC) in order to assess the additional performance of these imaging biomarkers, although such a comparison may be conservative as certain imaging biomarkers may be an early indicator of these conventional tumor attributes.

Use of anonymized data for academic purposes was approved by the Regional Ethical Committee Uppsala, Örebro, Sweden (Dnr 2008/081).

## 3. Results 

We had data on 1,346 women diagnosed with invasive breast cancer from 1996 to 1998 (n=498, the test cohort) and 2006 to 2010 (n=848, the validation cohort). The median follow-up time was 12.43 years for women diagnosed between 1996 and 1998 and 6.97 years for women diagnosed between 2006 and 2010. All women are Caucasians.  Table 1 shows the demographics, mammographic appearance of the tumor, basal phenotype, histologic tumor distribution, conventional tumor attributes, type of surgery, and adjuvant therapy (radiotherapy, chemotherapy, and Tamoxifen) in the two cohorts.

### 3.1. Long-Term Breast Cancer Survival by Imaging Biomarkers

**Figure 1:**
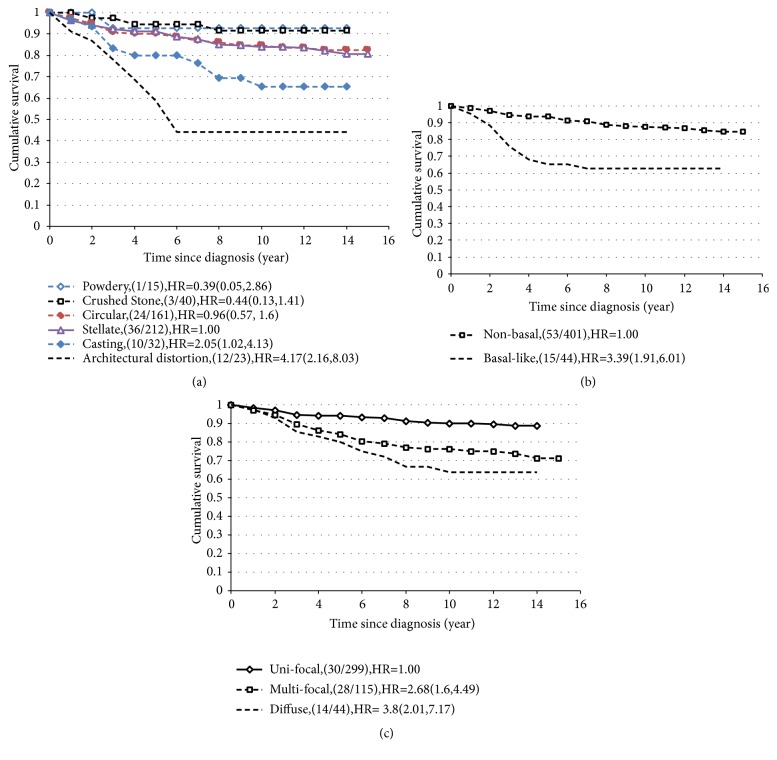
Cumulative 14-year survival of invasive breast cancers diagnosed in Falun 1996-1998. (a) According to mammographic tumor features. (b) According to basal phenotype. (c) According to histologic tumor distribution.

The 14-year cumulative survival curves by mammographic appearance of the tumor, basal phenotype, and histologic tumor distribution status are shown in Figures 1(a)–1(c). The survival curves by mammographic appearance of the tumor (Figure 1(a)) clearly illustrate that the prognosis was most favorable for tumors with powdery calcifications (92.9%) and the crushed stone-like type (91.7%), followed by the stellate (80.6%) and the circular types (82.6%); the casting type was associated with poor survival (65.4%), and the survival rate for the architectural distortion type (44.1%) was extremely poor. The difference in the 14-year survival across the six appearance types was statistically significant (p<0.0001). As seen in Figure 1(b), 14-year survival was poorer for the basal phenotype compared with the nonbasal phenotype (62.5% versus 84.6%; p<0.0001). Fourteen-year survival results differed significantly (p<0.0001) by histologic tumor distribution: diffuse type, 63.9%; multifocal type, 71.2%; and unifocal type, 88.8% (Figure 1(c)).

### 3.2. Associations between Imaging Biomarkers

The associations between mammographic tumor appearance and each of the other predictors, including basal phenotype, histologic tumor distribution, and conventional tumor attributes, are presented in Table 2. We combined three mammographic tumor appearance features (stellate tumors, powdery calcifications, and crushed stone-like calcifications on the mammogram) as noted above. Breast tumors with a circular appearance on mammography were more likely to have the basal phenotype (22.0%) compared with tumors showing the other appearance types (architectural distortion, 16.7%; casting-type calcifications 11.9%; and stellate tumors, powdery calcifications, or crushed stone-like calcifications on the mammogram 3.1%).

The association between mammographic tumor features and the histologic tumor distribution is striking. More than 70% of the casting-type tumors and those with architecture distortion were diffuse and had poor survival. Conversely, most circular and stellate/powdery/crushed stone-like types, which had good survival rates, were unifocal.

The mammographic tumor appearance was also associated with conventional tumor attributes (tumor size, histologic malignancy grade, and positive lymph nodes). Circular and stellate/powdery/crushed stone-like types were associated with smaller, node-negative, and well-differentiated breast tumors. The similar results based on the test cohort alone are specified in Table S1.

The associations between basal phenotype and other predictors using the test cohort are specified in Table S2. Basal phenotype and histologic tumor distribution were not significantly associated. Compared with the nonbasal phenotype, the basal phenotype was associated with larger, poorly differentiated breast tumors, and node-positive tumors.  Table S3 presents the relationships between histologic tumor distribution and the other predictors using the test cohort. Large, node-positive, and poorly differentiated breast tumors were observed in both the multifocal and diffuse breast cancers, showing an association between histologic tumor distribution and conventional tumor attributes. Tables S4-S6 show the similar results based on the validation cohort.

### 3.3. Assessment of Independent Effect

Table 3 gives the adjusted and unadjusted hazard ratios for breast cancer death and 95% confidence intervals (CI), with the estimated results for the three main variables of interest in the usual time order of clinical assessment. In the unadjusted (crude) analysis, the appearance of architectural distortion and the presence of casting type calcifications on the mammogram were strong predictors of breast cancer death, conferring 4.73-fold (95% CI: 2.48–9.05) and 2.33-fold (95% CI:1.17–4.66) increased risks, respectively, compared with the stellate/powdery/crushed stone-like calcification group. The basal phenotype was associated with a greater risk of breast cancer death than the nonbasal phenotype (HR=3.39; 95% CI: 1.91–6.01). The risk for breast cancer death was 3.80-fold for diffuse tumors and 2.68-fold for multifocal tumors compared with unifocal tumors.

After adjustment for conventional tumor attributes, type of surgery, and adjuvant therapy (Table 3), mammographic tumor appearance remained an independent predictor of breast cancer death (p=0.0003); the presence of casting-type calcifications and architectural distortion on the mammogram was associated with 3.13-fold and 3.19-fold risks, respectively; and the basal phenotype independently conferred 2.68-fold risk compared with the nonbasal phenotype (p=0.0058). Since the effect of diffuse histologic tumor distribution on survival was captured by tumors with casting-type calcifications and architectural distortion, we did not include the effect of the diffuse status in the final multivariable regression model to avoid colinearity. The HR of circular-type basal tumors was clearly affected by the incorporation of tumor size, node status, type of surgery, and adjuvant therapy.

Tables S7-S8 also present the comparison of the results between the trained cohort and the validated cohort. The results of the univariate analysis on the crude hazard ratio (HR) from both cohorts are very similar. Note that the effect sizes of treatment and therapy were affected by the period effect and were included when the predicted model was constructed by using the validation cohort. However, the effect sizes on the results of multivariate analysis based on the validation cohort were attenuated due to insufficient statistical power as the follow-up time of the validation cohort is too short to have sufficient numbers of breast cancer deaths. The validation dataset is therefore tailored for validation rather than training parameters.

### 3.4. New Classification of Risk Group

**Figure 2:**
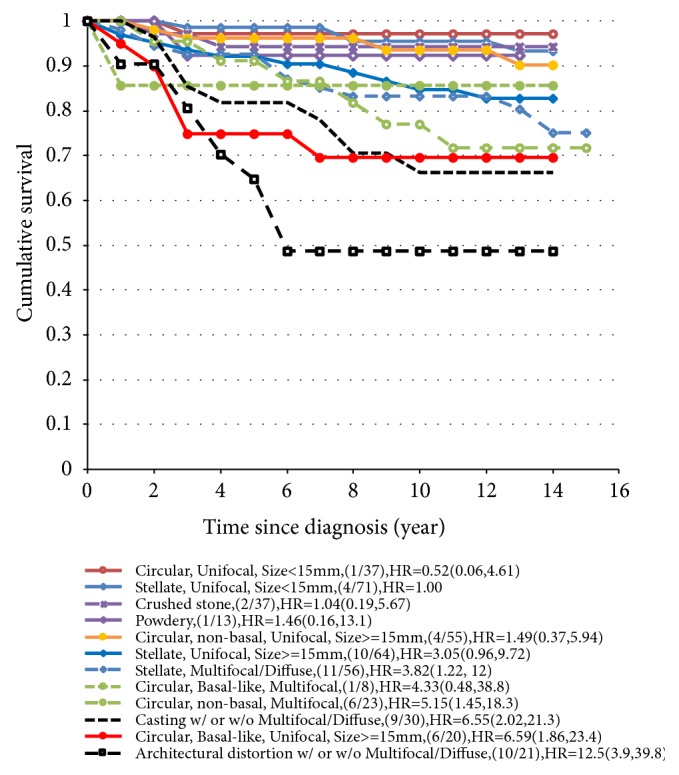
Cumulative survival of invasive breast cancer diagnosed in Falun 1996-1998, according to mammographic appearance, tumor size, histologic tumor distribution, and basal phenotype.

**Figure 3:**
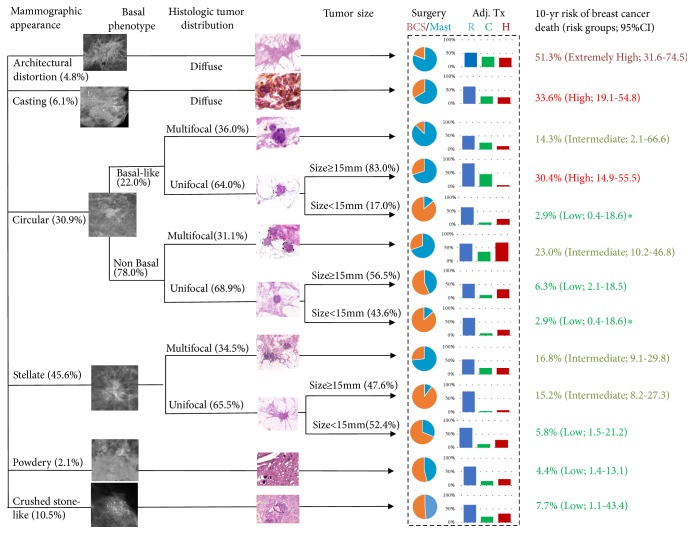
Combining mammographic appearance, histologic tumor distribution, and molecular subtype where relevant to classify cases into risk groups and 10-year breast cancer death. BCS, breast conserving surgery; Mast, mastectomy; Adj Tx, adjuvant therapy; R, radiotherapy; C, chemotherapy; H, Hormone therapy.

By combining mammographic tumor appearance, basal phenotype, tumor size, and histologic tumor distribution, 12 classification categories of risk profiles for breast cancer death were created (Figure 2). A 10-year risk of breast cancer death of less than 10% is defined as the low-risk group; an 11%-24% risk is the intermediate-risk group; a 25%–40% risk is the high-risk group; and a greater than 40% risk is defined as an extremely high-risk group (Figure 3). Note that Figure 3 also shows how surgical types (conservative surgery and mastectomy) and adjuvant therapies (radiotherapy, chemotherapy, and hormone therapy) were driven by the combination of three-dimensional imaging biomarkers as shown in the box surrounded by the dotted line in Figure 3. For example, the lower the risk of breast cancer death is (such as small circular unifocal tumors with nonbasal phenotype), the more likely the patient will undergo conservative surgery and will be less likely to undergo chemotherapy.

### 3.5. Validation

The observed breast cancer deaths in the validation cohort in 12 risk categories did not differ significantly from those predicted by the model (p=0.44). The lack of statistical significance suggests that the model is valid. The comparison between the observed and the expected values based on the validation cohort by risk groups has been given in Table S8. The observed and the expected values in different time periods by risk groups also have been plotted in Figure S1. The discrepancy is very small. However, as numbers of breast cancer are sparse, such a comparison should be taken with great caution. Compared with the model that only included conventional tumor attributes, the AUC of ROC increased from 77% to 84% when the mammographic appearance, basal phenotype, and histological tumor distribution was added to the model.

## 4. Discussion 

Early detection through mammography screening results in a significantly reduced incidence rate of large, node-positive, and poorly differentiated tumors. However, conventional tumor attributes do not readily distinguish the small fraction of cancers that potentially are lethal from the larger fraction that are not. While there has been a growing presence of critical commentary in the literature about overtreatment, a considerable volume of overtreatment occurs among women with small, favorable prognosis cancers. Given the fact that breast cancer treatments often are associated with short and long-term morbidity that can significantly diminish quality of life and functional status, avoiding overly aggressive treatment when it is not needed should be a high priority [29]. Avoiding undertreatment is no less important. Thus, there is a critical need for new indicators to refine the breast cancer risk profiles to choose more optimal treatment modalities.

We combined mammographic tumor appearance, basal phenotype, and histologic tumor distribution to identify a classification of risk profiles to predict breast cancer death. The risk profiles are based on data from a long-term longitudinal follow-up of 498 invasive breast cancer cases, and this classification was validated using a new independent dataset. These risk profiles can be grouped into 4 categories based on mammographic appearance, basal phenotype, and the combination of histologic tumor distribution and tumor size: the lowest risk group (≤ 10%) consists of stellate unifocal breast tumors <15mm in size, tumors with powdery and crushed stone-like calcifications, circular nonbasal phenotype unifocal tumors, and circular basal phenotype unifocal tumors <15mm in size; the intermediate risk group (11-24%) consists of stellate unifocal tumors ≥15mm in size, breast tumors with stellate multifocal appearance, and circular (basal and nonbasal phenotype) multifocal tumors; the high risk group (25-40%) consists of circular unifocal basil phenotype tumors ≥15 mm in size, and tumors with casting-type calcifications on the mammogram; and the extremely high risk group > 40% presents with architectural distortion without associated calcifications on the mammogram. These risk groups, shown in Figure 3, supplement the role of conventional tumor attributes.

Tumors with casting-type calcifications or architectural distortion without associated calcifications on the mammogram showed the poorest survival regardless of basal phenotype or histologic tumor distribution. However, few basal phenotype tumors were accompanied by calcifications of any kind, with the majority typically being circular masses. Three mammographic tumor appearance types, stellate tumors without calcifications and tumors with powdery and crushed stone-like calcifications with or without associated tumor mass on the mammogram, had favorable survival rates irrespective of basal phenotype or histologic tumor distribution. Circular-appearing tumors with a nonbasal phenotype and unifocal status showed lower risk of breast cancer death (2.9% for small tumors; 6.3% for large tumors) than those with nonbasal phenotype and multifocal appearance (23.0% 10-year risk). For unifocal tumors with basal phenotype, patient survival was strongly dependent on the tumor size: 10-year risk of death was 2.9% for a tumor < 15 mm, and 30.4% for a tumor ≥ 15 mm.

Mammographic tumor appearance usually is the first available breast cancer prognostic indicator, and these data suggest that mammographic tumor appearance combined with other indicators would be useful for treatment planning. Breast tumors with powdery and crushed stone-like mammographic tumor appearance (see Figure 3) may indicate a lower risk, and treatment and therapy provided for these cases may not need to be aggressive. In contrast, tumors with casting-type calcifications or architectural distortion, regardless of size, are very aggressive breast tumors. The potential for early detection of these tumors by mammography may be limited, and at the time of diagnosis both types require the use of magnetic resonance imaging (MRI) and ultrasonography for determining the full extent of breast tissue involved. These tumors also will benefit from large-section histology assessment, extensive surgery, and regular, intensive follow-up. Although these tumors represent a small proportion of cases, they are disproportionately responsible for a larger proportion of breast cancer deaths [5]. In this series, casting type tumors accounted for 6% of the invasive breast cancers, but 12% of the breast cancer deaths.

Given these observations, it may be unnecessary to test for the basal phenotype in patients with powdery, stellate, or crushed stone-like appearance tumors because it is so rare. However, testing for the basal phenotype is indicated for circular-type tumors, in addition to tumors with a circular/oval-shaped appearance on the mammogram, as shown previously by Luck et al. who also demonstrated that screen-detected breast tumors with the basal phenotype were more likely to reveal mammographic tumor appearance features of an ill-defined mass compared with breast tumors having the nonbasal phenotype [30].

The basal phenotype elevated the risk for breast cancer death 2.68-fold, even after adjusting for mammographic tumor appearance and tumor attributes. However, for breast cancer detected at a very early stage when tumors measure < 15 mm, prognosis is favorable, even for the basal phenotype. The difference in survival between basal and nonbasal phenotype tumors is observed in larger tumors, as the basal phenotype is more predictive of breast cancer death in tumors ≥ 15mm. This not only emphasizes the need for strict adherence to screening intervals to detect the more aggressive basal phenotype tumor at an early stage but also affects the choice of treatment modalities. Referring to Figure 3, among the basal phenotype unifocal circular lesions < 15 mm, the 10-year risk of death is 2.9% compared with 30.4% for the lesions ≥ 15 mm. Given the fact that conventional approaches to treatment planning lead to similar therapy for women with a unifocal tumor with the basal phenotype regardless of tumor size, these data suggest that the need for aggressive treatment should be reconsidered if the tumor size is small.

Determining histological tumor distribution is important for tumors of all mammographic tumor appearance types, particularly for stellate type and circular nonbasal types as they account for 46% and 24% of the breast cancers. Figure 3 shows that circular, unifocal, and nonbasal tumors are a low risk group regardless of tumor size and thus these patients also may not need aggressive treatment. Further, the risk of breast cancer death for stellate type breast cancer is highly dependent on histologic tumor distribution. The one-third of these tumors that are unifocal and size <15mm may not need aggressive treatment, whereas the one-third with larger size or multifocality needs complete surgical removal, and thus use of MRI to specify the extent of disease should be considered to insure optimal surgical treatment and adjuvant therapies as appropriate.

There are two main concerns over the methodology. First, the validation design used here is with external validation that is more reliable and stringent than the conventional cross-validation (the test cohort with part of the samples and the validated cohort with the rest of samples) as the external cohort is independent of the test cohort. However, the main limitation is that the follow-up time of the validation cohort is too short to have sufficient numbers of breast cancer death. However, similar findings were noted on the comparison of the test cohort and the validated cohort in the univariate analysis (Table S1-S8), and also the comparison of the expected and the observed survival curves in different time periods by risk groups (Figure S1) also was similar. A larger external validation cohort is therefore required to verify the proposed new classification. Moreover, the effect of treatment and therapy should not be taken as one part of the attribute-based predictors, but rather decision factors determined by surgeons and oncologists (see the last two column of Figure 3) and could vary with time periods due to the advance in treatment. This period effect should be taken into account into the model, when different periods are considered, for predicting the prognosis but not for risk classification.

The second is that other causes of death are considered as independent censorship rather than informative censorship. To relieve this concern, we analyzed the data with the competing risk model. The results are very similar between the competing risk model and the conventional survival model that treated other causes of death as censored cases (Table S9). The only difference is that the use of the competing risk model may affect the statistical power of some variables such as the basal phenotype.

There is the third limitation related to the problem of collinearity between basal phenotype and histologic tumor distribution when they were assessed as independent prognostic predictors. Due to limited sample size, we needed to combine three tumor categories (the stellate, powdery calcifications, and crushed stone-like calcification) as one group. In the future, it will be preferable to analyze these three categories separately in a larger study.

## 5. Conclusions

The combined information regarding mammographic tumor features, histologic tumor distribution and basal phenotype enables us to distinguish groups with different degrees of risk of breast cancer death. The model sequentially incorporates different dimensional attributes according to the chronological order in which the information is obtained. The combined imaging biomarker information for risk classification may be relevant for determining the need for molecular testing or other investigations, planning treatment, and determining the most appropriate clinical surveillance schedule for breast cancer patients. Our prognostic model was validated on an independent dataset. However, we believe further validation or refinement using larger tumor series is required and should be pursued, particularly, to elucidate intercorrelational but complementary role of imaging biomarkers.

## Figures and Tables

**Table 1:** Distribution of age, mammographic appearance, histologic tumor distribution, and conventional tumor attributes in two study cohorts.

	Women diagnosed between 1996-1998		Women diagnosed between 2006-2010	
	Number	Percentage (%)	Number	Percentage (%)
*Age, years*				
<40	13	2.61	21	2.48
40–49	67	13.45	117	13.80
50–59	113	22.69	169	19.93
60–69	113	22.69	254	29.95
70–79	107	21.49	171	20.17
80+	85	17.07	116	13.68
*Mammographic appearance*				
Stellate	212	43.89	371	46.55
Circular	161	33.33	235	29.49
Powdery	15	3.11	12	1.51
Casting	32	6.63	46	5.77
Crushed stone-like	40	8.28	94	11.79
Architectural distortion	23	4.76	39	4.89
NK	15		51	
*Basal type*				
Non-basal phenotype	401	90.11	634	88.80
Basal phenotype	44	9.89	80	11.20
NK	53		134	
*Histologic tumor distribution*				
Unifocal	299	65.28	458	55.25
Multifocal	115	25.11	301	36.31
Diffuse	44	9.61	70	8.44
NK	40		19	
*Tumor size (mm)*				
1–9	85	18.52	152	18.51
10–14	92	20.04	192	23.39
15–19	95	20.70	178	21.68
20–29	114	24.84	187	22.78
30–49	58	12.64	88	10.72
50+	15	3.27	24	2.92
NK	39		27	
*Histologic malignancy grade*				
I/II	341	74.13	627	76.84
III	119	25.87	189	23.16
NK	38		32	
*Lymph node involvement*				
Negative	331	72.43	513	61.51
Positive	126	27.57	321	38.49
NK	41		14	
*Surgery type*				
Breast conserving surgery	252	54.55	309	50.32
Mastectomy	210	45.45	305	49.68
NK	36		234	
*Radiotherapy*				
No	195	39.47	206	34.39
Yes	299	60.53	393	65.61
NK	4		249	
*Chemotherapy*				
No	408	82.59	382	63.88
Yes	86	17.41	216	36.12
NK	4		250	
*Hormone therapy*				
No	374	75.71	207	34.62
Yes	120	24.29	391	65.38
NK	4		250	

NK, not known.

**Table 2:** Relationship of mammographic appearance to basal phenotype, histologic tumor distribution, and conventional tumor attributes in both cohorts combined.

	Stellate/Powdery/Crushed stone-like	Circular	Casting	Architectural distortion	NK	*χ* ^2^ *∗*	P value
*Basal phenotype*						89.71	<0.0001
Non-basal	619 (96.87)	270 (78.03)	59 (88.06)	45 (83.33)	42		
Basal	20 (3.13)	76 (21.97)	8 (11.94)	9 (16.67)	11		
NK	105	50	11	8	13		
*Histologic tumor distribution*						817.74	<0.0001
Unifocal	450 (63.11)	260 (68.78)	7 (9.21)	7 (11.48)	33		
Multifocal	257 (36.04)	116 (30.69)	12 (15.79)	9 (14.75)	22		
Diffuse	6 (0.84)	2 (0.53)	57 (75.00)	45 (73.77)	4		
NK	31	18	2	1	7		
*Tumor size (mm)*						53.96	<0.0001
<15	340 (47.82)	127 (33.78)	31 (42.47)	3 (5.00)	20		
≥15	371 (52.18)	249 (66.22)	42 (57.53)	57 (95.00)	40		
NK	33	20	5	2	6		
*Histologic malignancy grade*						128.08	<0.0001
I/II	613 (86.46)	227 (60.21)	35 (48.61)	53 (86.89)	40		
III	96 (13.54)	150 (39.79)	37 (51.39)	8 (13.11)	17		
NK	35	19	6	1	9		
*Lymph node involvement*						22.52	<0.0001
Negative	479 (66.81)	258 (68.07)	45 (59.21)	23 (38.33)	39		
Positive	238 (33.19)	121 (31.93)	31 (40.79)	37 (61.67)	20		
NK	27	17	2	2	7		

*∗* Those with unknown mammography feature were not included in the chi-square test. NK, not known.

**Table 3:** Unadjusted (crude HR) and adjusted hazard ratios (aHR) for the effects of mammographic appearance, basal phenotype, histologic tumor distribution, conventional tumor attributes, surgery, and adjuvant therapy on the risk for breast cancer death.

Characteristic	No. of alive or OCD (%)	No. of BCD (%)	Crude HR (95%CI)	aHR (95%CI)
*Mammographic appearance*				
Stellate/Powdery/Crushed stone-like	227 (85.02)	40 (14.98)	1.00	1.00
Circular	137 (85.09)	24 (14.91)	1.09 (0.66, 1.80)	0.82 (0.48, 1.39)
Casting	22 (68.75)	10 (31.25)	2.33 (1.17, 4.66)	3.13 (1.46, 6.70)
Architectural distortion	11 (47.83)	12 (52.17)	4.73 (2.48, 9.05)	3.19 (1.55, 6.56)
*Basal phenotype*				
Non-basal	348 (86.78)	53 (13.22)	1.00	1.00
Basal	29 (65.91)	15 (34.09)	3.39 (1.91, 6.01)	2.68 (1.33, 5.39)
*Histologic tumor distribution*				
Unifocal	269 (89.97)	30 (10.03)	1.00	1.00
Multifocal	87 (75.65)	28 (24.35)	2.68 (1.60, 4.49)	1.62 (0.95, 2.76)
Diffuse	30 (68.18)	14 (31.82)	3.80 (2.01, 7.17)	–
*Tumor size (mm)*				
<15	165 (93.22)	12 (6.78)	1.00	1.00
≥15	222 (78.72)	60 (21.28)	3.77 (2.03, 7.02)	1.01† (1.00, 1.02)
*Histologic malignancy grade*				
I/II	298 (87.39)	43 (12.61)	1.00	1.00
III	89 (74.79)	30 (25.21)	2.28 (1.43, 3.63)	1.10 (0.65, 1.86)
*Lymph node involvement*				
Negative	298 (90.03)	33 (9.97)	1.00	1.00
Positive	86 (68.25)	40 (31.75)	3.90 (2.46, 6.19)	3.04 (1.76, 5.27)
*Surgery type*				
Breast conserving surgery	251 (87.15)	37 (12.85)	1.00	1.00
Mastectomy	155 (73.81)	55 (26.19)	2.39 (1.58, 3.63)	1.10 (0.63, 1.91)
*Radiotherapy*				
No	153 (78.46)	42 (21.54)	1.00	1.00
Yes	250 (83.61)	49 (16.39)	0.59 (0.39, 0.89)	0.59 (0.36, 0.97)
*Chemotherapy*				
No	342 (83.82)	66 (16.18)	1.00	1.00
Yes	61 (70.93)	25 (29.07)	1.74 (1.10, 2.75)	0.64 (0.36, 1.13)
*Hormone therapy*				
No	311 (83.16)	63 (16.84)	1.00	1.00
Yes	92 (76.67)	28 (23.33)	1.52 (0.97, 2.37)	1.15 (0.71, 1.87)

† size was used as a continuous variable.

OCD, other causes of death; BCD, breast cancer death; CI, confidence interval.

## Data Availability

The data used to support the findings of this study are available from the corresponding author upon request.

## Additional Points

This paper was done by The Swedish Two County Trial Research Group. The members of The Swedish Two County Trial Research Group are presented in the authors' list in the front matter of the paper.

## Supplementary Materials

Supplementary Materials Supplementary materials contain relationships between mammographic appearance and biomarkers (Tables S1-S6), the estimated results of Cox regression model in both test and validation cohorts (Tables S7), the estimated results of Cox regression model with and without consideration of competing risk of death (Table S9), and model validation with observed and expected numbers by risk groups (Table S8 and Figure S1). The table and figure legends are as follows. Table S1: relationship of mammographic appearance to basal phenotype, histologic tumor distribution, and conventional tumor attributes in test cohort. Table S2: relationship between basal phenotype and mammographic appearance, histologic tumor distribution, and tumor attributes in the test cohort. Table S3: relationship between histologic tumor distribution and mammographic appearance, basal phenotype, and tumor attributes in the test cohort. Table S4: relationship of mammographic appearance to basal phenotype, histologic tumor distribution, and conventional tumor attributes in validation cohort. Table S5: relationship between basal phenotype and mammographic appearance, histologic tumor distribution, and tumor attributes in the validation cohort. Table S6: relationship between histologic tumor distribution and mammographic appearance, basal phenotype, and tumor attributes in the validation cohort. Table S7: unadjusted (crude HR) and adjusted hazard ratios (aHR) for the effects of mammographic appearance, basal phenotype, histologic tumor distribution, and conventional tumor attributes on the risk for breast cancer death by test and validation cohort. Table S8: the observed and expected number of breast cancer death (BCD) by four risk groups. Table S9: results of univariate and multivariable Cox regression models before and after considering the competing risk of death in the test cohort. Figure S1: the observed (O) and expected (E) survival curve by four risk groups. (a) Low risk, (b) intermediate risk, (c) high risk, and (d) extremely high risk.
