# Supplementary material for: Imaging Biomarkers as Predictors for Breast Cancer Death
Source: J Oncol. 2019 Apr 10;2019:2087983. doi: 10.1155/2019/2087983 (PMC6481030; doi:10.1155/2019/2087983)
Supplement: Supplementary Materials — contain relationships between mammographic appearance and biomarkers (Tables S1-S6), the estimated results of Cox regression model in both test and validation cohorts (Tables S7), the estimated results of Cox regression model with and without consideration of competing risk of death (Table S9), and model validation with observed and expected numbers by risk groups (Table S8 and Figure S1). The table and figure legends are as follows. Table S1: relationship of mammographic appearance to basal phenotype, histologic tumor distribution, and conventional tumor attributes in test cohort. Table S2: relationship between basal phenotype and mammographic appearance, histologic tumor distribution, and tumor attributes in the test cohort. Table S3: relationship between histologic tumor distribution and mammographic appearance, basal phenotype, and tumor attributes in the test cohort. Table S4: relationship of mammographic appearance to basal phenotype, histologic tumor distribution, and conventional tumor attributes in validation cohort. Table S5: relationship between basal phenotype and mammographic appearance, histologic tumor distribution, and tumor attributes in the validation cohort. Table S6: relationship between histologic tumor distribution and mammographic appearance, basal phenotype, and tumor attributes in the validation cohort. Table S7: unadjusted (crude HR) and adjusted hazard ratios (aHR) for the effects of mammographic appearance, basal phenotype, histologic tumor distribution, and conventional tumor attributes on the risk for breast cancer death by test and validation cohort. Table S8: the observed and expected number of breast cancer death (BCD) by four risk groups. Table S9: results of univariate and multivariable Cox regression models before and after considering the competing risk of death in the test cohort. Figure S1: the observed (O) and expected (E) survival curve by four risk groups. (a) Low risk, (b) intermediate risk, (c) high [file 2087983.f1.docx]

Table S1. Relationship of mammographic appearance to basal phenotype, histologic tumor distribution, and conventional tumor attributes in test cohort

|  | Stellate/Powdery/Crushed stone-like | Circular | Casting | Architectural distortion | NK | χ^2^* | P value |
| --- | --- | --- | --- | --- | --- | --- | --- |
| **Basal phenotype** | |  |  |  |  | 40.25 | <0.0001 |
| Non-basal | 235 (97.51) | 113 (78.47) | 29 (96.67) | 18 (85.71) | 6 |  |  |
| Basal | 6 (2.49) | 31 (21.53) | 1 (3.33) | 3 (14.29) | 3 |  |  |
| NK | 26 | 17 | 11 | 2 | 6 |  |  |
| **Histologic tumor distribution** | | |  |  |  | 312.88 | <0.0001 |
| Unifocal | 171 (68.67) | 114 (78.08) | 3 (9.68) | 4 (18.18) | 7 |  |  |
| Multifocal | 77 (30.92) | 30 (20.55) | 2 (6.45) | 4 (18.18) | 2 |  |  |
| Diffuse | 1 (0.40) | 2 (1.37) | 26 (83.87) | 14 (63.64) | 1 |  |  |
| NK | 18 | 15 | 1 | 1 | 5 |  |  |
| **Tumor size (mm)** | |  |  |  |  | 16.32 | 0.001 |
| <15 | 114 (45.60) | 47 (32.19) | 10 (32.26) | 2 (9.09) | 4 |  |  |
| ≥15 | 136 (54.40) | 99 (67.81) | 21 (67.74) | 20 (90.91) | 6 |  |  |
| NK | 17 | 15 | 1 | 1 | 5 |  |  |
| **Histologic malignancy grade** | | |  |  |  | 45.77 | <0.0001 |
| I/II | 214 (85.60) | 87 (59.18) | 15 (48.39) | 18 (81.82) | 7 |  |  |
| III | 36 (14.40) | 60 (40.82) | 16 (51.61) | 4 (18.18) | 3 |  |  |
| NK | 17 | 14 | 1 | 1 | 5 |  |  |
| **Lymph node involvement** | |  |  |  |  | 6.25 | 0.0999 |
| Negative | 189 (75.90) | 102 (69.86) | 22 (70.97) | 11 (52.38) | 7 |  |  |
| Positive | 60 (24.10) | 44 (30.14) | 9 (29.03) | 10 (47.62) | 3 |  |  |
| NK | 18 | 15 | 1 | 2 | 5 |  |  |

Table S2. Relationship between basal phenotype and mammographic appearance, histologic tumor distribution, and tumor attributes in the test cohort

|  | Non-basal | Basal | χ^2^ | P value |
| --- | --- | --- | --- | --- |
| **Mammographic appearance** |  |  | 40.25 | <0.0001 |
| Stellate/Powdery/Crushed stone-like | 235 (59.49) | 6 (14.63) |  |  |
| Circular | 113 (28.61) | 31 (75.61) |  |  |
| Casting | 29 (7.34) | 1 (2.44) |  |  |
| Architectural distortion | 18 (4.56) | 3 (7.32) |  |  |
| NK | 6 | 3 |  |  |
| **Histologic tumor distribution** |  |  | 3.22 | 0.2002 |
| Unifocal | 261 (65.09) | 29 (67.44) |  |  |
| Multifocal | 98 (24.44) | 13 (30.23) |  |  |
| Diffuse | 42 (10.47) | 1 (2.33) |  |  |
| NK | 0 | 1 |  |  |
| **Tumor Size (mm)** |  |  | 9.58 | 0.0020 |
| <15 | 162 (40.40) | 7 (16.28) |  |  |
| ≥15 | 239 (59.60) | 36 (83.72) |  |  |
| NK |  |  |  |  |
| **Histologic malignancy grade** |  |  | 82.93 | <0.0001 |
| I/II | 324 (81.00) | 8 (18.18) |  |  |
| III | 76 (19.00) | 36 (81.82) |  |  |
| NK |  |  |  |  |
| **Lymph node involvement** |  |  | 14.70 | 0.0001 |
| Negative | 297 (75.00) | 21 (47.73) |  |  |
| Positive | 99 (25.00) | 23 (52.27) |  |  |
| NK | 5 | 0 |  |  |

Table S3. Relationship between histologic tumor distribution and mammographic appearance, basal phenotype, and tumor attributes in the test cohort

|  | Unifocal | Multifocal | Diffuse | χ^2^ | P value |
| --- | --- | --- | --- | --- | --- |
| **Mammographic appearance** |  |  |  | 312.88 | <0.0001 |
| Stellate/powdery/crushed stone-like | 171 (58.56) | 77 (68.14) | 1 (2.33) |  |  |
| Circular | 114 (39.04) | 30 (26.55) | 2 (4.65) |  |  |
| Casting | 3 (1.03) | 2 (1.77) | 26 (60.47) |  |  |
| Architectural distortion | 4 (1.37) | 4 (3.54) | 14 (32.56) |  |  |
| NK | 7 | 2 | 1 |  |  |
| **Basal phenotype** |  |  |  | 3.22 | 0.2002 |
| Non-basal | 261 (90.00) | 98 (88.29) | 42 (97.67) |  |  |
| Basal | 29 (10.00) | 13 (11.71) | 1 (2.33) |  |  |
| NK | 9 | 4 | 1 |  |  |
| **Tumor Size (mm)** |  |  |  | 13.71 | 0.0011 |
| <15 | 133 (44.48) | 35 (30.43) | 9 (20.45) |  |  |
| ≥15 | 166 (55.52) | 80 (69.57) | 35 (79.55) |  |  |
| **Histologic malignancy grade** |  |  |  | 1.06 | 0.5898 |
| I/II | 222 (74.25) | 88 (77.88) | 31 (70.45) |  |  |
| III | 77 (25.75) | 25 (22.12) | 13 (29.55) |  |  |
| NK | 0 | 2 | 0 |  |  |
| **Lymph node involvement** |  |  |  | 25.06 | <0.0001 |
| Negative | 236 (80.00) | 65 (57.02) | 26 (60.47) |  |  |
| Positive | 59 (20.00) | 49 (42.98) | 17 (39.53) |  |  |
| NK | 4 | 1 | 1 |  |  |

Table S4. Relationship of mammographic appearance to basal phenotype, histologic tumor distribution, and conventional tumor attributes in validation cohort

|  | Stellate/Powdery/Crushed stone-like | Circular | Casting | Architectural distortion | NK | χ^2^* | P value |
| --- | --- | --- | --- | --- | --- | --- | --- |
| **Basal phenotype** | |  |  |  |  | 54.16 | <0.0001 |
| Non-basal | 384 (96.48) | 157 (77.72) | 30 (81.08) | 27 (81.82) | 36 |  |  |
| Basal | 14 (3.52) | 45 (22.28) | 7 (18.92) | 6 (18.18) | 8 |  |  |
| NK | 79 | 33 | 37 | 6 | 7 |  |  |
| **Histologic tumor distribution** | | |  |  |  | 516.23 | <0.0001 |
| Unifocal | 279 (60.13) | 146 (62.93) | 4 (8.89) | 3 (7.69) | 26 |  |  |
| Multifocal | 180 (38.79) | 86 (37.07) | 10 (22.22) | 5 (12.82) | 20 |  |  |
| Diffuse | 5 (1.08) | 0 (0.00) | 31 (68.89) | 31 (79.49) | 3 |  |  |
| NK | 13 | 3 | 1 | 0 | 2 |  |  |
| **Tumor size (mm)** | |  |  |  |  | 39.31 | <0.0001 |
| <15 | 226 (49.02) | 80 (34.78) | 21 (50.00) | 1 (2.63) | 16 |  |  |
| ≥15 | 235 (50.98) | 150 (65.22) | 21 (50.00) | 37 (97.37) | 34 |  |  |
| NK | 16 | 5 | 4 | 1 | 1 |  |  |
| **Histologic malignancy grade** | | |  |  |  | 81.93 | <0.0001 |
| I/II | 399 (86.93) | 140 (60.87) | 20 (48.78) | 35 (89.74) | 33 |  |  |
| III | 60 (13.07) | 90 (39.13) | 21 (51.22) | 4 (10.26) | 14 |  |  |
| NK | 18 | 5 | 5 | 0 | 4 |  |  |
| **Lymph node involvement** | |  |  |  |  | 20.51 | 0.0001 |
| Negative | 290 (61.97) | 156 (66.95) | 23 (51.11) | 12 (30.77) | 32 |  |  |
| Positive | 178 (38.03) | 77 (33.05) | 22 (48.89) | 27 (69.23) | 17 |  |  |
| NK | 9 | 2 | 1 | 0 | 2 |  |  |

Table S5. Relationship between basal phenotype and mammographic appearance, histologic tumor distribution, and tumor attributes in the validation cohort

|  | Non-basal | Basal | χ^2^ | P value |
| --- | --- | --- | --- | --- |
| **Mammographic appearance** |  |  | 54.16 | <0.0001 |
| Stellate/Powdery/Crushed stone-like | 384 (64.21) | 14 (19.44) |  |  |
| Circular | 157 (26.25) | 45 (62.50) |  |  |
| Casting | 30 (5.02) | 7 (9.72) |  |  |
| Architectural distortion | 27 (4.52) | 6 (8.33) |  |  |
| NK | 36 | 8 |  |  |
| **Histologic tumor distribution** |  |  | 1.27 | 0.5309 |
| Unifocal | 342 (53.94) | 45 (56.25) |  |  |
| Multifocal | 237 (37.38) | 31 (38.75) |  |  |
| Diffuse | 55 (8.68) | 4 (5.00) |  |  |
| **Tumor Size (mm)** |  |  | 17.62 | <0.0001 |
| <15 | 277 (44.04) | 15 (19.23) |  |  |
| ≥15 | 352 (55.96) | 63 (80.77) |  |  |
| NK | 5 |  |  |  |
| **Histologic malignancy grade** |  |  | 114.53 | <0.0001 |
| I/II | 525 (83.60) | 24 (30.38) |  |  |
| III | 103 (16.40) | 55 (69.62) |  |  |
| NK | 6 |  |  |  |
| **Lymph node involvement** |  |  | 0.07 | 0.7983 |
| Negative | 397 (62.72) | 49 (61.25) |  |  |
| Positive | 263 (37.28) | 31 (38.75) |  |  |
| NK | 1 | 0 |  |  |

Table S6. Relationship between histologic tumor distribution and mammographic appearance, basal phenotype, and tumor attributes in the validation cohort

|  | Unifocal | Multifocal | Diffuse | χ^2^ | P value |
| --- | --- | --- | --- | --- | --- |
| **Mammographic appearance** |  |  |  | 516.23 | <0.0001 |
| Stellate/powdery/crushed stone-like | 279 (64.58) | 180 (64.06) | 5 (7.46) |  |  |
| Circular | 146 (33.80) | 86 (30.60) | 0 (0.00) |  |  |
| Casting | 4 (0.93) | 10 (3.56) | 31 (46.27) |  |  |
| Architectural distortion | 3 (0.69) | 5 (1.78) | 31 (46.27) |  |  |
| NK | 26 | 20 | 3 |  |  |
| **Basal phenotype** |  |  |  | 1.27 | 0.5309 |
| Non-basal | 342 (88.37) | 237 (88.43) | 55 (93.22) |  |  |
| Basal | 45 (11.63) | 31 (11.57) | 4 (6.78) |  |  |
| NK | 71 | 33 | 11 |  |  |
| **Tumor Size (mm)** |  |  |  | 25.21 | <0.0001 |
| <15 | 224 (49.34) | 104 (34.67) | 16 (24.24) |  |  |
| ≥15 | 230 (50.66) | 196 (65.33) | 50 (75.76) |  |  |
| NK | 4 | 1 | 4 |  |  |
| **Histologic malignancy grade** |  |  |  | 2.91 | 0.2335 |
| I/II | 356 (78.94) | 224 (74.92) | 47 (71.21) |  |  |
| III | 95 (21.06) | 75 (25.08) | 19 (28.79) |  |  |
| NK | 7 | 2 | 4 |  |  |
| **Lymph node involvement** |  |  |  | 62.63 | <0.0001 |
| Negative | 335 (73.30) | 140 (46.51) | 32 (45.71) |  |  |
| Positive | 122 (26.70) | 161 (53.49) | 38 (54.29) |  |  |
| NK | 1 | 0 | 0 |  |  |

Table S7. Unadjusted (crude HR) and adjusted hazard ratios (aHR) for the effects of mammographic appearance, basal phenotype, histologic tumor distribution, and conventional tumor attributes on the risk for breast cancer death by test and validation cohort.

|  | Test cohort | | | | Validation cohort | | | |
| --- | --- | --- | --- | --- | --- | --- | --- | --- |
| **Characteristic** | No. of alive or OCD (%) | No. of BCD (%) | Crude HR (95%CI) | aHR* (95%CI) | No. of alive or OCD (%) | No. of BCD (%) | Crude HR (95%CI) | aHR* (95%CI) |
| **Mammographic appearance** |  |  |  |  |  |  |  |  |
| Stellate/Powdery/Crushed stone-like | 227  (85.02) | 40  (14.98) | 1 | 1 | 455  (95.39) | 22  (4.61) | 1 | 1 |
| Circular | 137  (85.09) | 24  (14.91) | 1.09  (0.66, 1.80) | 0.82  (0.48, 1.39) | 224  (95.32) | 11  (4.68) | 1.07  (0.52, 2.21) | 0.51  (0.24, 1.06) |
| Casting | 22  (68.75) | 10  (31.25) | 2.33  (1.17, 4.66) | 3.13  (1.46, 6.70) | 41  (89.13) | 5  (10.87) | 2.52  (0.96, 6.66) | 1.11  (0.41, 2.99) |
| Architectural distortion | 11  (47.83) | 12  (52.17) | 4.73  (2.48, 9.05) | 3.19  (1.55, 6.56) | 32  (82.05) | 7  (17.95) | 4.25  (1.81, 9.95) | 1.22  (0.45, 3.33) |
| **Basal phenotype** |  |  |  |  |  |  |  |  |
| Non-basal | 348  (86.78) | 53  (13.22) | 1 | 1 | 601  (94.79) | 33  (5.21) | 1 | 1 |
| Basal | 29  (65.91) | 15  (34.09) | 3.39  (1.91,6.01) | 2.68  (1.33, 5.39) | 70  (87.50) | 10  (12.50) | 2.62  (1.29, 5.31) | 1.16  (0.50, 2.70) |
| **Histologic tumor distribution** |  |  |  |  |  |  |  |  |
| Unifocal | 269  (89.97) | 30  (10.03) | 1 | 1 | 443  (96.72) | 15  (3.28) | 1 | 1 |
| Multifocal | 87  (75.65) | 28  (24.35) | 2.68  (1.60,4.49) | 1.62  (0.95, 2.76) | 276  (91.69) | 25  (8.31) | 2.59  (1.37,4.92) | 1.31  (0.73, 2.37) |
| Diffuse | 30  (68.18) | 14  (31.82) | 3.8  (2.01,7.17) | ̶ | 59  (84.29) | 11  (15.71) | 5.21  (2.39, 11.35) | ̶ |
| **Tumor size (mm)** |  |  |  |  |  |  |  |  |
| <15 | 165  (93.22) | 12  (6.78) | 1 | 1 | 337  (97.97) | 7  (2.03) | 1 | 1 |
| ≥15 | 222  (78.72) | 60  (21.28) | 3.77  (2.03, 7.02) | 1.01†  (1.00, 1.02) | 432  (90.57) | 45  (9.43) | 5.21  (2.35, 11.56) | 1.04†  (1.02, 1.05) |
| **Histologic malignancy grade** |  |  |  |  |  |  |  |  |
| I/II | 298  (87.39) | 43  (12.61) | 1 | 1 | 602  (96.01) | 25  (3.99) | 1 | 1 |
| III | 89  (74.79) | 30  (25.21) | 2.28  (1.43, 3.63) | 1.1  (0.65, 1.86) | 164  (86.77) | 25  (13.23) | 3.57  (2.05, 6.21) | 2.39  (1.29, 4.41) |
| **Lymph node involvement** |  |  |  |  |  |  |  |  |
| Negative | 298  (90.03) | 33  (9.97) | 1 | 1 | 498  (97.08) | 15  (2.92) | 1 | 1 |
| Positive | 86  (68.25) | 40  (31.75) | 3.9  (2.46,6.19) | 3.04  (1.76, 5.27) | 285  (88.79) | 36  (11.21) | 3.88  (2.13, 7.09) | 1.57  (0.83, 2.97) |

*Model adjusted for the surgery type, and adjuvant therapy

† size was used as a continuous variable

OCD, other cause of death; BCD, breast cancer death

Table S8. The observed and expected number of breast cancer death
(BCD) by four risk groups

| Risk group | No. of subjects | Observed BCD | Expected BCD |
| --- | --- | --- | --- |
| Low | 277 | 10 | 7.59 |
| Intermediate | 280 | 13 | 18.03 |
| High | 55 | 5 | 7.92 |
| Extremely High | 31 | 6 | 7.31 |

Table S9. Results of univariate and multivariable Cox regression models before and after considering the competing risk of death in the test cohort

|  | Without adjustment | | Adjusting for competing risk of death | |
| --- | --- | --- | --- | --- |
| **Characteristic** | Crude HR | aHR* | Crude HR | aHR* |
|  | (95%CI) | (95%CI) | (95%CI) | (95%CI) |
| **Age** |  |  |  |  |
| increasing 1 year | 1.03 | 1.02 | 1.01 | 1.01 |
|  | (1.01, 1.04) | (1.01, 1.04) | (1.00, 1.03) | (0.99, 1.03) |
| **Mammographic appearance** |  |  |  |  |
| Stellate/Powdery/Crushed stone-like | 1.00 | 1.00 | 1.00 | 1.00 |
| Circular | 1.09 | 0.78 | 1.00 | 0.82 |
|  | (0.66, 1.80) | (0.46, 1.33) | (0.60, 1.66) | (0.47, 1.43) |
| Casting | 2.33 | 3.59 | 2.27 | 2.89 |
|  | (1.17, 4.66) | (1.67, 7.71) | (1.15, 4.48) | (1.31, 6.40) |
| Architectural distortion | 4.73 | 3.32 | 4.59 | 3.5 |
|  | (2.48, 9.05) | (1.62, 6.80) | (2.40, 8.76) | (1.71, 7.17) |
| **Basal phenotype** |  |  |  |  |
| Non-basal | 1.00 | 1.00 | 1.00 | 1.00 |
| Basal | 3.39 | 2.62 | 3.16 | 2.03 |
|  | (1.91,6.01) | (1.30, 5.26) | (1.74, 5.74) | (0.94, 4.41) |
| **Histologic tumor distribution** |  |  |  |  |
| Unifocal | 1.00 | 1.00 | 1.00 | 1.00 |
| Multifocal | 2.68 | 1.90 | 2.64 | 1.70 |
|  | (1.60,4.49) | (1.11, 3.23) | (1.58,4.41) | (0.97, 2.99) |
| Diffuse | 3.8 | ̶ | 3.58 | ̶ |
|  | (2.01,7.17) |  | (1.91, 6.73) |  |
| **Tumor size (mm)** |  |  |  |  |
| <15 | 1.00 | 1.00 | 1.00 | 1.00 |
| ≥15 | 3.77 | 1.01† | 3.47 | 1.01† |
|  | (2.03, 7.02) | (1.00, 1.02) | (1.88, 6.39) | (1.00, 1.02) |
| **Histologic malignancy grade** |  |  |  |  |
| I/II | 1.00 | 1.00 | 1.00 | 1.00 |
| III | 2.28 | 1.17 | 2.21 | 1.18 |
|  | (1.43, 3.63) | (0.69, 1.99) | (1.39, 3.54) | (0.68, 2.03) |
| **Lymph node involvement** |  |  |  |  |
| Negative | 1.00 | 1.00 | 1.00 | 1.00 |
| Positive | 3.90 | 2.92 | 3.71 | 2.14 |
|  | (2.46, 6.19) | (1.67, 5.09) | (2.34, 5.87) | (1.25, 3.68) |

*Model adjusted for the surgery type, and adjuvant therapy

HR: hazard ratio, aHR: adjusted hazard ratio

Figure S1. The observed (O) and expected (E) survival curve by four risk groups. (a) low risk, (b) intermediate risk, (c) high risk and (d) extremely high risk

|  |  |
| --- | --- |
|  |  |
